# Invasiveness of plants is predicted by size and fecundity in the native range

**DOI:** 10.1002/ece3.1432

**Published:** 2015-04-17

**Authors:** Kim Jelbert, Iain Stott, Robbie A McDonald, Dave Hodgson

**Affiliations:** 1 Environment and Sustainability Institute, University of ExeterPenryn Campus, Penryn, Cornwall TR10 9FE, UK; 2 Centre for Ecology and Conservation, University of ExeterPenryn Campus, Penryn, Cornwall TR10 9FE, UK

**Keywords:** Basal stem diameter, demography, fecundity, invasive, native, phylogeny, plant

## Abstract

1. An important goal for invasive species research is to find key traits of species that predispose them to being invasive outside their native range.
2. Comparative studies have revealed phenotypic and demographic traits that correlate with invasiveness among plants. However, all but a few previous studies have been performed in the invaded range, an approach which potentially conflates predictors of invasiveness with changes that happen during the invasion process itself.
3. Here, we focus on wild plants in their native range to compare life-history traits of species known to be invasive elsewhere, with their exported but noninvasive relatives. Specifically, we test four hypotheses: that invasive plant species (1) are larger; (2) are more fecund; (3) exhibit higher fecundity for a given size; and (4) attempt to make seed more frequently, than their noninvasive relatives in the native range. We control for the effects of environment and phylogeny using sympatric congeneric or confamilial pairs in the native range.
4. We find that invasive species are larger than noninvasive relatives. Greater size yields greater fecundity, but we also find that invasives are more fecund per-unit-size.
5. *Synthesis*: We provide the first multispecies, taxonomically controlled comparison of size, and fecundity of invasive versus noninvasive plants in their native range. We find that invasive species are bigger, and produce more seeds, even when we account for their differences in size. Our findings demonstrate that invasive plant species are likely to be invasive as a result of both greater size and constitutively higher fecundity. This suggests that size and fecundity, relative to related species, could be used to predict which plants should be quarantined.

## Introduction

Invasive species consistently rank among the five major threats to biodiversity, worldwide (Sala et al. 2000; Butchart et al. 2010; Kareiva and Marvier 2011) and are costly to the global economy (Pimentel et al. 2005). Given the economic (Pimentel et al. 2005) and ecological costs of invasive plant species (Vilà et al. 2011), it is unsurprising that considerable attention has been given to understanding the characteristics (Rejmánek and Richardson 1996; Ramula et al. 2008; Pyšek et al. 2009; Burns et al. 2013) and the underlying mechanisms associated with invasion success (Prentis et al. 2008; Davidson et al. 2011). The many traits and mechanisms thought to influence invasiveness have been reviewed extensively elsewhere (Pyšek and Richardson 2007; Prentis et al. 2008; van Kleunen et al. 2010; Davidson et al. 2011); here, we focus on the demographic traits of size and fecundity, first because they provide a close link between phenotype, life history, and population dynamics (Stott et al. 2011) and second because, if shown to be markers of invasiveness, they are relatively simple to measure in the field.

As postulated by Baker (1965), it is intuitive that invasive species will be more fecund and grow faster than noninvasive species. Fast growth and large size may afford introduced species an advantage over the floristic assemblage of the invaded environment. Evidence for this comes from studies that have shown invasive species to (1) be larger than their native or noninvasive introduced counterparts (Reichard and Hamilton 1997; Pyšek and Richardson 2007; van Kleunen et al. 2010); (2) grow faster than their noninvasive congeners in the invaded range (Grotkopp et al. 2002; Burns 2006); and (3) grow faster and attain a larger size (biomass, root: shoot ratio, and leaf length) than their noninvasive congeners in the native range (van Kleunen et al. 2011). However, Burns (2006) found that invasive species of Commelinaceae were not significantly larger than their noninvasive congeners. Fecundity has also been identified as an important correlate of invasiveness in the invaded range (Burns 2006; Mason et al. 2008; Moravcová et al. 2010; Burns et al. 2013). Propagule pressure (the number of seeds or viable clonal material reaching a new site) is an important correlate of invasiveness (Holle and Simberloff 2005); therefore, more fecund individuals or species can be assumed to have greater opportunity to colonize new sites (Westoby et al. 2002). However, evidence for this is both conflicting and surprisingly scarce. We attribute this to the paucity of fecundity data in field guides, which form a typical source of data for comparative analyses of traits associated with invasiveness. In the invaded range, invasive species have been shown to exhibit higher fecundity than (1) their introduced, noninvasive congeners (Burns 2006; Burns et al. 2013), (2) noninvasive, introduced, unrelated species (Moravcová et al. 2010), and (3) native species (Mason et al. 2008). However, conversely, Daehler (2003) found that of thirteen comparisons of invasive–native confamilial pairings in the invaded range, invasive species had no consistent reproductive advantage over co-occurring natives.

These approaches, while enormously valuable, have three weaknesses that limit their suitability for identifying predictors of invasiveness:

1. All, with the exception of van Kleunen and Johnson (2007); Schlaepfer et al. (2010); van Kleunen et al. (2011), are performed in the invaded range, an approach which conflates predictors of invasiveness with changes that may happen during the invasion process. Of the studies performed in the native range (van Kleunen and Johnson 2007; Schlaepfer et al. 2010; van Kleunen et al. 2011), none consider fecundity as a potential correlate of invasiveness. Environmental variation is known to contribute to significant variation in demographic parameters and predictions (Morris and Doak 2005; Buckley et al. 2010). Measuring demographic parameters, such as fecundity, in the invaded range is therefore a measure following change induced by the novel environment. We suggest that demographic parameters associated with invasiveness in the invaded range may be poor predictors of invasiveness, when the objective is to identify potential invaders prior to their introduction.
2. All but one study (van Kleunen et al. 2010) compare invasive species with species that are native or noninvasive at the study location only: several of these native or “noninvasive” species are known to be invasive elsewhere. If invasive species share an “invasiveness” trait or syndrome, we should expect comparisons with species that are invasive elsewhere to mask or weaken potential correlates of invasiveness.
3. None considers the effect of plant size on fecundity. Plant size is critical because we know that within a species, larger individuals typically exhibit higher fecundity (Weiner et al. 2009) and because increased plant height, larger specific leaf area (Grotkopp et al. 2002; Pyšek and Richardson 2007) and biomass (Schlaepfer et al. 2010; van Kleunen et al. 2011) have been identified as correlates of invasiveness. This raises an important question: Are invasive plant species invasive because they are larger and therefore more fecund, or because they exhibit a constitutively higher fecundity, that is. higher fecundity per-unit-size, than their noninvasive counterparts?

Here, we focus on traits expressed by wild plants in their native range, and compare them between species that are invasive elsewhere, and species that are established elsewhere but not invasive. We control for the effects of phylogeny using congener/confamilial pairs (Burns et al. 2013). We also control for environmental effects by studying sympatric populations in a restricted geographical zone (mid and west Cornwall, UK). We hypothesize that invasive plants (1) are larger than their native, noninvasive relatives; (2) are therefore more fecund; (3) but for a given size, exhibit higher fecundity, and (4) attempt to make seed more frequently than their native, noninvasive relatives. To our knowledge, this is the first study to investigate fecundity in the native range as a predictor of invasiveness. This novel approach accounts for the potential effects of phylogeny, environment, and global invasive status and has the potential to identify true differences in life-history parameters (in this instance size and fecundity) between invasive and noninvasive species.

## Materials and Methods

### Species

Five sympatric congener/confamilial pairs of plant species (Table1) were selected on the basis that each pair (1) comprised one native species that is invasive elsewhere and one native species that is introduced but noninvasive elsewhere, (2) occurred sympatrically in the native range, (3) comprised accessible and sufficiently large populations to facilitate monitoring, and (4) represented a broad range of angiosperm families. Where possible, species pairs (5) occupied a similar geographical native range, and f) belonged to the same life-form (i.e., perennial or annual; herb or shrub).

**Table 1:** Species pairs: life-form, breeding system, status, and mean seed production per inflorescence

Family	Species	Common name	Life-Form	Breeding System^1^	Mean seed production per inflorescence	Status	Citation
Caryophyllaceae	*Cerastium fontanum*	Common mouse–ear	Per	Hermaphrodite; protoandrous; automatic self or cross	52	Invasive	USDA; ISSG
	*Cerastium diffusum*	Sea mouse–ear	Ann	Hermaphrodite; automatic self	19	Introduced	USDA
Caryophyllaceae	*Silene dioica*	Red campion	Per	Dioecious; obligatory cross	277	Invasive	Jenkins and Keller (2011); CABI; Randall (2012)^2^
	*Silene uniflora*	Sea campion	Per	Gynodioecious; protoandrous; automatic self or cross	57	Introduced	CHAH
Ericaceae	*Calluna vulgaris*	Heather	Shrub	Hermaphrodite; weakly protoandrous; cross	8	Invasive	Australian Invasive Weed List; National Pest Plant Accord; ISSG
	*Erica cinerea*	Bell heather	Shrub	Hermaphrodite; weakly protoandrous; cross or automatic self	16	Introduced	CHAH
Scrophulariaceae	*Rhinanthus minor* subsp*. minor*	Yellow rattle	Ann	Hermaphrodite; automatic self or cross	11	Invasive	Hulst et al. (1987); CABI; Randall (2012)^2^
	*Pedicularis sylvatica*	Lousewort	Per	Hermaphrodite; cross	13	Introduced	USDA
Apiaceae	*Daucus carota*	Wild carrot	Per	Hermaphrodite; protoandrous; cross	934	Invasive	USDA
	*Eryngium maritimum*	Sea holly	Per	Hermaphrodite; protoandrous; cross	44	Introduced	USDA

1 Mating system derived from http://www.ecoflora.co.uk.

2 Invasive status based on number of citations in the GCWs (Randall 2012).

Plant status was determined by searching the Global Invasive Species Database (GISD) http://www.issg.org/database/welcome/, the Invasive Species Compendium (CABI) http://www.cabi.org/isc, the Australian Invasive Weed List http://www.environment.gov.au/biodiversity/invasive/weeds/weeds/lists/index.html, the Australian Plant Census (CHAH) http://www.anbg.gov.au/chah/apc/, the European and Mediterranean Plant Protection Organization (EPPO) database http://www.eppo.int/DATABASES/GD/gd.htm, Schedule 9 of the Wildlife and Countryside Act (1981) http://jncc.defra.gov.uk/page-1377, the United States Department of Agriculture (USDA) Plant Database http://plants.usda.gov/checklist.html, the National Institute for Environmental Studies (NIES) invasive species of Japan database http://www.nies.go.jp/biodiversity/invasive, the National Pest Plant Accord http://www.biosecurity.govt.nz/nppa and using the following search term in Google “*Latin name* invasive” (accessed April 2013). Species are considered invasive when designated as “invasive” (also “weedy” or “noxious” in the USDA Plant Database) in one or more of the databases listed above or when designated as invasive by a Government Agency or Academic Institution. CABI cites two of our “invasive” congeners (*Silene dioica* and *Rhinanthus minor* subsp. *minor*) as invasive. While this status could not be verified from the CABI cited literature, both species are notoriously “weedy” (Hulst et al. 1987; Jenkins and Keller 2011) and have more citations in the Global Compendium of Weeds (GCWs) than their “noninvasive” congeners (Randall 2012). The GCWs collates citations referring to “weedy behavior” outside of the native range; the number of citations for each listed species has been used previously to determine global invasive status, and to successively identify correlates of invasiveness (Schlaepfer et al. 2010; Jenkins and Keller 2011). We therefore consider the designation of these species as “invasive” to be correct. A species was considered to be “introduced” if it was naturalized outside of its native range. A species was considered to be native to the UK if listed as such on the Online Atlas of the British and Irish Flora http://www.brc.ac.uk/plantatlas/.

### Location

Each study location (five in total: one for each species pair) was selected on the bases that it supported both species of each sympatric pair and that these populations could reliably be assumed to be native. To ensure that the sample populations were of native provenance, all sites were characterized by natural or seminatural vegetation, and sites were excluded where past and present management had the potential to have introduced plants of unknown provenance. Sites supporting sympatric species pairs were identified using the ERICA Database held by Dr. Colin French. ERICA, a database compiled by amateur and professional botanists, holds more than 1.3 million geo-referenced vascular plant records of the Cornish flora. To locate our sample populations, we produced co-incidence maps showing the 100 m distribution of each congener pair. Accessible sites were then ground-truthed to locate each sympatric population.

### Data collection

Permanently marked, geo-referenced quadrats were installed at each site. These were positioned in order to capture a representative sample of each sympatric population. Quadrat size was determined by the species’ area-weighted density and ranged from 0.5 × 0.5 m to 1 × 1 m. Larger species typically necessitated larger quadrats; however, within each species pair, quadrat size was the same. The number of quadrats sited per species ranged from eight to thirteen (mean - 10); this variation is a result of the species area-weighted density and abundance at the site. Each quadrat (permanently marked with buried metal chips) was made relocatable using a Global Positioning System (GPS) to provide a coarse location (accurate to within 10 m), and a metal detector to determine the exact location.

Individual plants within each quadrat were marked with colored, biodegradable, hemp string and were assigned a unique identification number corresponding to the individual's position within the quadrat. We consider an individual to be an entire plant or, for clonal rhizomatous species, a ramet (an individual belonging to a clonal group of genetically identical individuals) and use the term “plant(s)” interchangeably to refer to these individuals in this study. Using the physical markers and/or the unique identification code, it was possible to locate the same individuals repeatedly between May and November 2013, encompassing late spring, summer, and autumn. Each sample population was relocated on three occasions, the timing of which was determined by the reported plant life cycle and by interim visits. During each visit, we measured plant size (basal stem diameter, rosette diameter, and rosette perpendicular diameter) and recorded the life stage (i.e., seedling, vegetative, and reproductive) of all individuals within each quadrat. Basal stem diameter, defined as the diameter of the stem at ground level, was carefully measured to avoid damaging the plant, using 150, 0.1 mm precision, dialMax Vernier Dial Calipers. If present, we also recorded the number of seed capsules or racemes per plant (from which we calculated fecundity as described below). *Silene dioica* and to a lesser extent *Cerastium fontanum* were observed to exhibit a long flowering period lasting, in some instances, the duration of our study. For these species, the reported fecundity measure is considered conservative. Fortunately, both *Silene dioica* and *Cerastium fontanum* are invasive, and therefore a conservative measure will only favor the null hypothesis. The remaining eight species exhibit a comparatively short flowering period and do not set seed until flowering has ceased; reported fecundity is therefore considered an accurate measure of annual fecundity per individual.

In accordance with Burns et al. (2013), seed number was used to measure fecundity. To determine individual fecundity, the number of seed capsules/racemes per plant was counted. A representative sample of single seed capsules/racemes were collected from 30 individuals per species, and seeds counted using an Elmor C1 seed counter. The average number of seeds per fruit/raceme was then calculated. Individual fecundity was determined by multiplying average seed number per fruit/raceme by the number of fruits per plant.

### Data analysis

Exploratory analysis (mixed-effects model of log seed number against log basal stem diameter, rosette diameter, and rosette perpendicular diameter, with species identity as a random effect) revealed basal stem diameter to be the best correlate of fecundity for all species; we therefore used basal stem diameter to represent plant size in all subsequent data analysis. To determine whether invasive species were larger than their native noninvasive relatives, we used generalized linear mixed-effects models (GLMM) with “species pair” as a random effect, “species” and “quadrat” as nested random effects, “basal stem diameter” as a Gaussian response variable, and “invasive status” as a fixed effect. To determine whether invasive species were more fecund, we used the same modeling framework but with log-transformed seed number as the Gaussian-distributed response variable. The nesting of the random effects is crucial in this design: measures of size and fecundity for each individual plant are pseudoreplicates that contribute to the means for each species in each phylogenetic pair. The nested models correctly tested the influence of invasiveness on mean traits in each pair. To determine whether, for a given size, invasive species exhibit higher fecundity than their native noninvasive relatives, again we used the same modeling framework, but with “invasive status” and “basal stem diameter” as fixed effects. We tested the impact of invasive status and basal stem diameter on seed set, using likelihood ratio tests between models that included or excluded the “invasiveness” fixed effect.

To determine whether invasive species were more likely to make seed than their noninvasive relatives, we used the same modeling framework, but with “attempt to set seed” as a binary response variable: Each plant either flowered and produced seed, or did not. All analyses were performed using the lme4 package (Bates et al. 2014) in RStudio version 0.97.551 (R Core Team 2014). Model checks, following log-transformation of seed number and basal stem diameter, confirmed homoscedasticity and normality of standardized residuals in all analyses.

## Results

**Figure 1:**
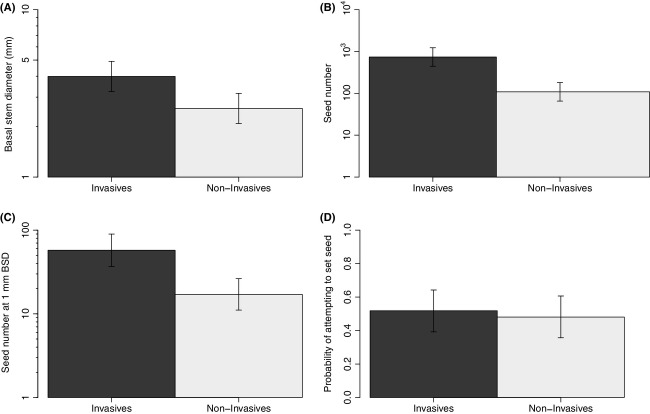
Differences in phenotypic and demographic traits between invasive and noninvasive species, in the native range. Bars show mean traits (± standard error bars) derived from hierarchical mixed-effects models, controlling for phylogenetic pairing and averaged across pseudoreplicates within species. (A) basal stem diameter for invasive (black bar) and noninvasive (gray bar) species; (B) seed number for invasive and noninvasive species; (C) seed number for invasive and noninvasive species at a 1 mm basal stem diameter (BSD); and (D) probability of invasive and noninvasive species attempting to set seed. The *y*-axis of figure 1a–1c is on a log scale.

**Figure 2:**
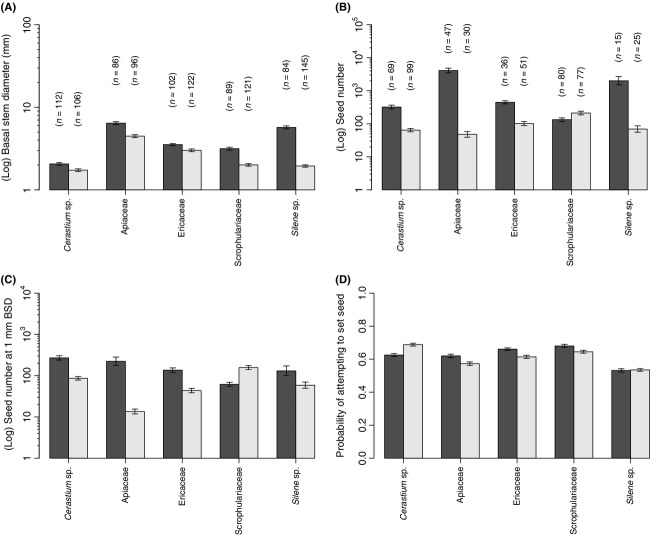
Differences in phenotypic and demographic traits between invasive and noninvasive species, in the native range. Bars show mean traits (± standard error bars) for each species; “*n*” represents the number of individuals sampled. (A) mean (log) basal stem diameter for invasive (black bar) and noninvasive (gray bar) species within each congener/confamilial pair; (B) mean (log) seed number for invasive and noninvasive species within each congener/confamilial pair; (C) mean (log) seed number (fecundity) for invasive and noninvasive species within each congener/confamilial pair at a 1 mm basal stem diameter; and (D) probability of invasive and noninvasive species within each congener/confamilial pair attempting to set seed. The *y*-axis of figure 2A–2C is on a log scale.

Invasive species had significantly larger basal stem diameters than their noninvasive relatives (χ^2^ - 4.4487, df - 1, *P *-* *0.035) (Fig.1A). All pairs exhibited this relationship (Fig.2A).

**Figure 3:**
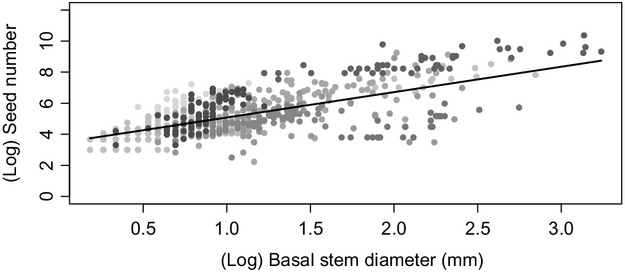
The relationship between basal stem diameter and fecundity. Points represent measurements of individual plants. Members of each confamilial pair share the same grayscale shading. Fitted line represents a common slope across species and a single intercept for the “average” species.

Across all species basal stem diameter was positively correlated with fecundity (χ^2^ - 230.62, df - 1, *P *<* *0.001) (Fig.3). We found that invasive species exhibit significantly higher fecundity than their noninvasive relatives (χ^2^ - 6.3753, df - 1, *P *-* *0.012 (Fig.1B). We also found that invasive species exhibit significantly higher fecundity per-unit-size than their noninvasive relatives (χ^2^ - 4.2286, df - 1, *P *-* *0.039; Fig.1C). When considering the raw data, four of five of our congener/confamilial pairs exhibited this relationship (Fig.2B,C). The fifth confamilial pair (Scrophulariaceae) did not fit the overall pattern: For a given basal stem diameter, the noninvasive species *Pedicularis sylvatica* exhibited higher fecundity than its invasive relative *Rhinanthus minor* subsp. *minor* (Fig.2C). Note, however, that a greater proportion of the population of the invasive *R. minor* subsp. *minor* set seed (Fig.2D).

Finally, we found that, across phylogenetic pairs, invasive species do not attempt to make seed more frequently than their native, noninvasive relatives (χ^2^ - 0.1726, df - 1, *P *-* *0.678; Fig.1D).

## Discussion

Biological plant invasions are both economically (Pimentel et al. 2005) and ecologically costly (Vilà et al. 2011), and so there is considerable impetus to identify predictors of invasiveness. By measuring fecundity, size, and population structure for five sympatric congener/confamilial pairs in the native range, we tested four hypotheses: that invasive plant species (1) are larger; (2) are more fecund; (3) exhibit higher fecundity for a given size; and (4) attempt to make seed more frequently, than their noninvasive relatives in the native range.

We confirmed three of our four hypotheses; invasive plant species are larger, more fecund, and more fecund per-unit-size, than their native, noninvasive relatives. Hypothesis 4 was rejected: invasive species do not attempt to make seed more frequently than their native, noninvasive relatives. Our findings, and those of comparative studies in the invaded range (Burns 2006; Mason et al. 2008; Moravcová et al. 2010; Burns et al. 2013), support Baker's (1965) postulation that invasive/weedy species are likely to grow faster and be more fecund. However, unlike other comparative studies, we also considered the effect of plant size on fecundity. Plant size is important because we know that within a species, larger individuals typically exhibit higher fecundity (Weiner et al. 2009) and because increased plant height, larger specific leaf area (Grotkopp et al. 2002; Pyšek and Richardson 2007), and biomass (Schlaepfer et al. 2010; van Kleunen et al. 2011) have been identified as correlates of invasiveness. This raises an important question: Are invasive plant species invasive because they are larger (and therefore more fecund) or because they exhibit a constitutively higher fecundity (i.e., higher fecundity per-unit-size) than their noninvasive counterparts? We show that while invasive plant species are larger than their native, noninvasive relatives, invasives are also constitutively more fecund. Furthermore, we also consider the effect of population structure on fecundity. Population structure is important because a species exhibiting high individual fecundity but belonging to a population with few reproductive individuals may perform poorly in comparison to a species exhibiting lower individual fecundity but belonging to a population with many reproductive individuals. We find no evidence that invasive species attempt to make seed more frequently than their native noninvading relatives.

While our findings clearly demonstrate that invasive species are larger and exhibit constitutively higher fecundity than their native, non-invading relatives, there is an exception among our congener/confamilial pairs, which deserves discussion. Four of five pairs comprise an invasive species that exhibits higher fecundity than its noninvading relative. The only exception is the Scrophulariaceae pair for which the reverse is true: the noninvasive species *Pedicularis sylvatica* exhibits higher fecundity than its invasive relative *Rhinanthus minor* subsp. *minor*. One possible explanation pertains to the life-form of each congener. *Pedicularis sylvatica*, the noninvading species, is a perennial; therefore, while this species exhibits higher individual fecundity than its invasive congener, *R. minor* subsp. *minor,* the invasive congener is an annual that belongs to a population with a higher proportion of reproductive individuals in each growth season. The potential influence of plant breeding system on fecundity also deserves discussion. Several authors have identified autonomous seed production to be an important correlate of invasiveness (Rambuda and Johnson 2004; van Kleunen et al. 2008; Hao et al. 2011). High fecundity could therefore be correlated with a particular type of breeding system. However, among our congeners, a greater number of noninvasive species exhibit autonomous seed production; furthermore, within species pairs, there is considerable overlap in breeding system (Table1). This indicates that high fecundity is independent of breeding system in this study.

Comparative studies in the invaded range give three possible explanations for high fecundity as correlate of invasiveness (Mason et al. 2008; Burns et al. 2013): (1) invasives are able to increase allocation to seed production following release from natural enemies or competition; (2) invasives increase allocation to growth following release from natural enemies or competition, with a correlated increase in seed production; and (3) the invaded environment selects for introduced species with a constitutively high fecundity. Environmental variation contributes to significant variation in demographic parameters (Morris and Doak 2005; Buckley et al. 2010), therefore measuring demographic parameters in the invaded range cannot distinguish between constituent traits, or trait changes (caused by phenotypic plasticity or microevolution) that are induced by the novel environment. The same principle can be applied to phenotypic traits relating to size (Schlaepfer et al. 2010). Consequently, comparative studies in the invaded range (Mason et al. 2008; Burns et al. 2013) were unable to determine which explanation was most plausible. van Kleunen et al. (2011) and Schlaepfer et al. (2010) found that invasive species are larger (shoot: root ratio, leaf length and biomass) than their noninvasive relatives in the native range, indicating that larger species are more likely to be invasive. Our results support these findings: We show that invasive species are larger than their noninvasive relatives; however, uniquely, we show that invasive species are also constitutively more fecund than their noninvasive relatives in the native range, that is, prior to any change induced by the novel environment. Our findings suggest that the invaded environment is a biased filter that favors introduced species that are both large and constituently more fecund.

Propagule pressure has been identified as a correlate of invasiveness (Reichard and Hamilton 1997; Herron et al. 2007), and it seems probable that inter-regional propagule pressure (the number of dispersal units transported to a new region outside of the native range) is biased; some species are more likely to be transported than other species. We know that plant attractiveness is a correlate of invasiveness (Pyšek and Richardson 2007) so perhaps larger and more fecund plant species are more likely to be transported due to their esthetic qualities (i.e., inflorescence size) or functionality (i.e., robustness). Evidence for this comes from a positive correlation between inflorescence size and fecundity in the invasive plant *Silene latifolia* (Delph and Herlihy 2012) and from a study of South African Iridaceae. Among South African Iridaceae, a species is more likely to be naturalized if it is in horticultural use, and taller species are more likely to be used in horticulture (van Kleunen et al. 2007). Large size may also afford introduced species a competitive advantage over the existing floristic assemblage upon arrival.

The probability of a species colonizing a new site is assumed to increase with the number of dispersal units (seeds or clonal material) produced (Westoby et al. 2002). Evidence for this comes from a positive correlation between the number of seeds per plant, among naturalized *Crotalaria* species in Taiwan, and species frequency (Wu et al. 2005). More frequently occurring, and thus more “invasive” *Crotalaria* species, are more fecund than their less frequent, naturalized relatives (Wu et al. 2005). It therefore seems probable that more fecund species are more likely to be transported to a new region; and once present have a better capacity to spread rapidly due their ability to exert greater local propagule pressure (the number of dispersal units transported within the introduced range). High fecundity may also afford additional, more complex, advantages for invading species. The “perfect” invasive species is a species that colonizes fast, persists, and dominates at carrying capacity. Typical trade-offs of colonization and competitive ability are unlikely to be experienced by the “perfect” invasive species. Classic theory suggests that seed size (and by extension fecundity) is determined by the trade-off between competition and colonization (Turnbull et al. 1999). However, more recently Coomes et al. (2002) found that asymmetric competition of co-occurring annual forbs was insufficient to determine seed size; these authors suggest that variation in seed size is more likely to reflect a species’ ability to contract and expand its population in response to environmental conditions (Coomes et al. 2002; Coomes and Grubb 2003). Smaller seeded and therefore more fecund species, have a greater capacity to “boom and bust” (Stott et al. 2010) in response to environmental conditions and are typically more abundant than larger seeded, less fecund species (Coomes et al. 2002; Coomes and Grubb 2003). This suggests that more fecund species have a competitive advantage; however, understanding the relationship between high fecundity and population dynamics is less clear. The emerging study of transient dynamics (short-term dynamics of populations that are not at equilibrium) offers a potential explanation (Townley et al. 2007; Stott et al. 2011).

Transient dynamics of short-term boom and bust have been shown to be exaggerated among species with high fecundity (Stott et al. 2012). Furthermore, a comparative analysis of the transient population dynamics of 108 plant species identified that populations predicted to grow faster in the long-term exhibit greater potential magnitudes of transient amplification and attenuation (short-term increase and decrease respectively relative to asymptotic growth) than slower growing or declining populations (Stott et al. 2010). We know that amplification is linked to fecundity (Stott et al. 2012) and that invasive populations typically grow faster than native or noninvasive populations in the long term (Ramula et al. 2008; Burns et al. 2013). Therefore, perhaps the comparatively high fecundity of invasive populations compared to those of their native noninvading relatives reflects their greater propensity to amplify in the short term in response to exogenous disturbances, allowing them to colonize vacant niches quickly, coupled with faster population growth in the long term. This would be consistent with the observation that disturbed environments (those where exogenous disturbances occur more frequently) are more readily invaded than stable ones (D'Antonio et al. 1999; Marvier et al. 2004).

Our approach and findings are of direct relevance to the field of invasion biology and ecology. This is the first study to make interspecific comparisons of fecundity as a function of plant size and population structure among invasive/noninvasive congener and confamilial pairs that are representative of multiple life-forms. Furthermore, this study is the first to make such comparisons in the native range. Performance in the native range is very important because as invasion biologists/ecologists we are interested in identifying predictors of invasiveness. We know that environmental variation has potential to cause significant variation in demographic parameters and predictions (Morris and Doak 2005; Buckley et al. 2010); we therefore suggest that demographic parameters associated with invasiveness in the invaded range are poor predictors of invasiveness, when the objective is to identify potential invaders prior to their introduction.

We acknowledge that our study samples a small number of species pairs, in a restricted geographical area, during one plant growth season. Our findings might therefore be specific to the location of study and the plant assemblage present. Future work should establish whether our findings hold true for a greater number of phylogenetically paired species that are representative of multiple life-forms, and at a global scale. Future work should also test whether invasive populations, exhibiting high fecundity in the native range, grow faster in the long term than their sympatric, noninvasive, less fecund relatives; determine the importance of other demographic parameters in the growth and decline of invasive and noninvasive populations in the native range; and test the hypothesis that higher fecundity yields greater potential for both transient population amplification in response to disturbance, and long-term population growth.

## Data Accessibility

Data are lodged with Dryad add accession number and are available from the corresponding author on request.

## References

[b1] Baker HG (1965). Characteristics and modes of origin of weeds. The genetics of colonising species.

[b2] Bates D, Maechler M, Bolker B, Walker S (2014).

[b3] Buckley YM, Ramula S, Blomberg SP, Burns JH, Crone EE, Ehrlén J (2010). Causes and consequences of variation in plant population growth rate: a synthesis of matrix population models in a phylogenetic context. Ecol. Lett.

[b4] Burns JH (2006). Relatedness and environment affect traits associated with invasive and noninvasive introduced Commelinaceae. Ecol. Appl.

[b5] Burns JH, Pardini EA, Schutzenhofer MR, Chung YA, Seidler KJ, Knight TM (2013). Greater sexual reproduction contributes to differences in demography of invasive plants and their noninvasive relatives. Ecology.

[b6] Butchart SH, Walpole M, Collen B, van Strien A, Scharlemann JP, Almond RE (2010). Global biodiversity: indicators of recent declines. Science.

[b7] Coomes DA, Grubb PJ (2003). Colonization, tolerance, competition and seed-size variation within functional groups. Trends Ecol. Evol.

[b8] Coomes DA, Rees M, Grubb PJ, Turnbull L (2002). Are differences in seed mass among species important in structuring plant communities? Evidence from analyses of spatial and temporal variation in dune-annual populations. Oikos.

[b9] Daehler CC (2003). Performance comparisons of co-occurring native and alien invasive plants: implications for conservation and restoration. Annu. Rev. Ecol. Evol. Syst.

[b10] D'Antonio CM, Dudley TL, Mack M (1999). Disturbance and biological invasions: direct effects and feedbacks. In Walker L. ed. Ecosystems of Disturbed Ground.

[b11] Davidson AM, Jennions M, Nicotra AB (2011). Do invasive species show higher phenotypic plasticity than native species and, if so, is it adaptive? A meta-analysis. Ecol. Lett.

[b12] Delph LF, Herlihy CR (2012). Sexual, fecundity, and viability selection on flower size and number in a sexually dimorphic plant. Evolution.

[b13] Grotkopp E, Rejmánek M, Rost TL (2002). Toward a causal explanation of plant invasiveness: seedling growth and life-history strategies of 29 pine (Pinus) species. Am. Nat.

[b14] Hao JH, Qiang S, Chrobock T, van Kleunen M, Liu QQ (2011). A test of baker's law: breeding systems of invasive species of Asteraceae in China. Biol. Invasions.

[b15] Herron PM, Martine CT, Latimer AM, Leicht-Young SA (2007). Invasive plants and their ecological strategies: prediction and explanation of woody plant invasion in New England. Divers. Distrib.

[b16] Holle BV, Simberloff D (2005). Ecological resistance to biological invasion overwhelmed by propagule pressure. Ecology.

[b17] Hulst RV, Shipley B, Thériault A (1987). Why is *Rhinanthus minor* (Scrophulariaceae) such a good invader?. Can. J. Bot.

[b18] Jenkins C, Keller SR (2011). A phylogenetic comparative study of preadaptation for invasiveness in the genus Silene (Caryophyllaceae). Biol. Invasions.

[b19] Kareiva PM, Marvier M (2011). Conservation science: balancing the needs of people and nature.

[b20] van Kleunen M, Johnson SD (2007). South African Iridaceae with rapid and profuse seedling emergence are more likely to become naturalized in other regions. J. Ecol.

[b21] van Kleunen M, Johnson SD, Fischer M (2007). Predicting naturalization of southern African Iridaceae in other regions. J. Appl. Ecol.

[b22] van Kleunen M, Manning JC, Pasqualetto V, Johnson SD (2008). Phylogenetically independent associations between autonomous self-fertilization and plant invasiveness. Am. Nat.

[b23] van Kleunen M, Weber E, Fischer M (2010). A meta-analysis of trait differences between invasive and non-invasive plant species. Ecol. Lett.

[b24] van Kleunen M, Schlaepfer DR, Glaettli M, Fischer M (2011). Preadapted for invasiveness: do species traits or their plastic response to shading differ between invasive and non-invasive plant species in their native range?. J. Biogeogr.

[b25] Marvier M, Kareiva P, Neubert MG (2004). Habitat destruction, fragmentation, and disturbance promote invasion by habitat generalists in a multispecies metapopulation. Risk Anal.

[b26] Mason RAB, Cooke J, Moles AT, Leishman MR (2008). Reproductive output of invasive versus native plants. Glob. Ecol. Biogeogr.

[b27] Moravcová L, Pyšek P, Jarošík V, Havlíčková V, Zákravský P (2010). Reproductive characteristics of neophytes in the Czech Republic: traits of invasive and non-invasive species. Preslia.

[b28] Morris WF, Doak DF (2005). How general are the determinants of the stochastic population growth rate across nearby sites?. Ecol. Monogr.

[b29] Pimentel D, Zuniga R, Morrison D (2005). Update on the environmental and economic costs associated with alien-invasive species in the United States. Ecol. Econ.

[b30] Prentis PJ, Wilson JRU, Dormontt EE, Richardson DM, Lowe AJ (2008). Adaptive evolution in invasive species. Trends Plant Sci.

[b31] Pyšek P, Nentwig W, Richardson DM (2007). Traits associated with invasiveness in alien plants: where do we stand?. Ecological studies.

[b32] Pyšek P, Křivánek M, Jarošík V (2009). Planting intensity, residence time, and species traits determine invasion success of alien woody species. Ecology.

[b33] R Core Team (2014). R: a language and environment for statistical computing.

[b34] Rambuda TD, Johnson SD (2004). Breeding systems of invasive alien plants in South Africa: does Baker's rule apply?. Divers. Distrib.

[b35] Ramula S, Knight TM, Burns JH, Buckley YM (2008). General guidelines for invasive plant management based on comparative demography of invasive and native plant populations. J. Appl. Ecol.

[b36] Randall RP (2012). A global compendium of weeds.

[b37] Reichard SH, Hamilton CW (1997). Predicting invasions of woody plants introduced into North America. Conserv. Biol.

[b38] Rejmánek M, Richardson DM (1996). What attributes make some plant species more invasive?. Ecology.

[b39] Sala OE, Chapin FS, Armesto JJ, Berlow E, Bloomfield J, Dirzo R (2000). Global biodiversity scenarios for the year 2100. Science.

[b40] Schlaepfer DR, Glaettli M, Fischer M, van Kleunen M (2010). A multi-species experiment in their native range indicates pre-adaptation of invasive alien plant species. New Phytol.

[b41] Stott I, Franco M, Carslake D, Townley S, Hodgson D (2010). Boom or bust? A comparative analysis of transient population dynamics in plants. J. Ecol.

[b42] Stott I, Townley S, Hodgson DJ (2011). A framework for studying transient dynamics of population projection matrix models. Ecol. Lett.

[b43] Stott I, Hodgson DJ, Townley S (2012). Beyond sensitivity: nonlinear perturbation analysis of transient dynamics. Methods Ecol. Evol.

[b44] Townley S, Carslake D, Kellie-Smith O, McCarthy D, Hodgson D (2007). Predicting transient amplification in perturbed ecological systems. J. Appl. Ecol.

[b45] Turnbull LA, Rees M, Crawley MJ (1999). Seed mass and the competition/colonization trade-off: a sowing experiment. J. Ecol.

[b46] Vilà M, Espinar JL, Hejda M, Hulme PE, Jarošík V, Maron JL (2011). Ecological impacts of invasive alien plants: a meta-analysis of their effects on species, communities and ecosystems. Ecol. Lett.

[b47] Weiner J, Campbell LG, Pino J, Echarte L (2009). The allometry of reproduction within plant populations. J. Ecol.

[b48] Westoby M, Falster DS, Moles AT, Vesk PA, Wright IJ (2002). Plant ecological strategies: some leading dimensions of variation between species. Annu. Rev. Ecol. Syst.

[b49] Wu S-H, Rejmánek M, Grotkopp E, DiTomaso JM (2005). Herbarium records, actual distribution, and critical attributes of invasive plants: genus Crotalaria in Taiwan. Taxon.

